# Sustainable Business Model in the Product-Service System: Analysis of Global Research and Associated EU Legislation

**DOI:** 10.3390/ijerph181910123

**Published:** 2021-09-26

**Authors:** Ana Batlles-delaFuente, Luis Jesús Belmonte-Ureña, José Antonio Plaza-Úbeda, Emilio Abad-Segura

**Affiliations:** 1 Department of Economy and Business, University of Almeria, 04120 Almeria, Spain; anabatlles@ual.es (A.B.-d.); lbelmont@ual.es (L.J.B.-U.); japlaza@ual.es (J.A.P.-Ú.); 2 Research Centre CIAMBITAL, University of Almeria, 04120 Almeria, Spain

**Keywords:** product-service system, servitisation, circular economy, sustainability, bibliometric, systematic review

## Abstract

The business fabric is trying to resolve the many transformations that have occurred in recent decades. Companies are obliged to offer new ways to meet the needs of the market. This situation has led to the creation of new business models that combine both competitiveness and sustainability. Among the most consistent strategies, the product-service system (PSS) stands out. A bibliometric analysis was carried out on 1088 documents during the period 2000–2020, to synthesize the knowledge base on PSS in a global context and analyse future trends. The results obtained have made it possible to identify the evolution of scientific production, the main drivers of this issue, the lines of research developed and their link with EU legislation and reveal some critical gaps in knowledge. The main lines of research describe different aspects of PSS: servitisation, product design, manufacturing, life cycle, circular economy, and sustainable development. This study has identified how its analysis has developed to date and what terms allow us to glimpse new approaches; hence, it is a useful tool for PSS researchers and sponsors who provide financial resources that allow new directions in this research.

## 1. Introduction

Nowadays, the business world is trying to resolve the numerous transformations that have been taking place in recent decades. Companies are obliged to offer new ways to meet market needs, mainly derived from events such as excessive competition, the market full of products, the information that the consumer retains transferred into greater demand, in addition to the continuous depletion of the natural resources. All these reasons have led to the creation of new business models that combine competitiveness and sustainability [1,2,3]. 

Among the most consistent strategies, the one that involves services in the acquisition process in order to satisfy current consumer demands stands out, that is, the product-service system (PSS) [4,5]. It is true that companies have always offered services together with products, but in recent decades there has been a greater methodical approach integrating them. Consequently, the growing trend for the study of systems that comprise products and services has emerged from different areas, such as the environment and marketing [6].

In this context, the PSS results from strategic innovation from modifying the traditional business approach of designing, manufacturing and selling products, when selling the system of products and services that meets the specific needs of consumers [7,8]. Furthermore, the introduction of PSS in the market is arguable, because the economic and social transformations of recent years have led to new clients and consumers, with a rethinking of consumption [9]. This will depend on the PSS offered, in terms of typology and product-service composition. On the one hand, clients may be interested in receiving services in addition to the product, but it always depends on the type of service offered and what the client must do to receive the service, such as sharing data. On the other hand, for the PSS provider, it is not simple to offer PSS since the efficient delivery of PSS often requires a reengineering of internal processes, which means dedicating time and effort that many companies are not interested in facing.

The motivation of this study is to find out how PSS research has evolved at an international level and its link with sustainability and the circular economy (CE). The review of the literature at a global level has allowed us to examine the works that address this topic, so that the research problem found on the PSS refers to determining: (i) what is the evolution of scientific production; (ii) what are the thematic areas to which the documents are associated; (iii) which are the most prolific journals, authors, institutions and countries; (iv) what are the thematic axes; and (v) what is the nature of the relationship with the legislation of the European Union (EU). Consequently, the objective of this work is to examine the dynamics of global research, during the period 2000–2020, on the PSS business model and related community legislation.

To obtain answers to the above research questions, a sample of 1088 scientific journal articles, books, and book chapters selected from the Scopus database (Elsevier) have been analysed. A bibliometric analysis has been carried out to synthesize the knowledge base on PSS in a global context. This methodology allows to know the status of the contributions on this subject made so far and, thus, update the lines of research.

The main limitations have been to check whether, among other factors, the number of published records is related to local or international criteria, stakeholder requirements in the decision-making process, or the development of new technologies applied to this business system.

The results obtained have made it possible to identify the evolution of scientific production, the main drivers of this issue, the lines of research developed and their link with EU legislation and reveal some critical knowledge gaps.

The main lines of research that have been developed during the analysed period (2000–2020) describe different aspects of the PSS: servitization, product design, manufacture, life cycle, CE, and sustainable development. This work is a useful tool for PSS researchers, since it has been identified how their analysis has developed to date and what terms allow us to glimpse new approaches, in addition to the sponsors who provide financial resources that allow new directions in this research.

This work is organized as follows. Section 2 develops the concept of PSS, the relationship with other concepts and their different approaches. Section 3 details the methodology applied. Section 4 shows the results obtained and their discussion in a broad context. Finally, Section 5 summarizes and concludes.

## 2. The Concept of Product-Service System: Literature Review

The concept of PSS arises in the late 1990s, and refers to a business system where a company offers a combination of services and products to satisfy a specific need of the client, instead of providing a single product that covers this function, that is, it provides services adding value to the product life cycle [10,11]. Fundamentally, it arises from the greater access to customer and end consumer information, which directly affects the characteristics of the demand. Likewise, the PSS is a beneficial solution for the producer, users and the environment, which is used in companies that contemplate improving the traditional way of obtaining resources, compete, generate value and social quality when consumption decreases [5,12]. 

Regarding the environmental variable, the PSS is limited to those systems that satisfy the needs and expectations of consumers more efficiently, with added value for the company and the consumer; In other words, it considers environmental logistics in a global way, referring to the set of sustainable policies and measures aimed at reducing the environmental impact caused by business activities. PSS can separate value creation from material and energy consumption and thus significantly reduce life-cycle environmental impact compared to traditional product-only systems [13,14].

The literature describes product-oriented PSS, when products are sold to the user, but additional services are added. The PSS is said to be usage-oriented when the business model is geared toward selling the product’s function. Finally, the PSS would be results-oriented, when the business model is aimed at selling a result and is the closest to offering a service, where it is not a predetermined product [15,16,17]. The main characteristics of the PSS include that (1) they associate products and services in a value proposition; (2) they pose a lucrative relationship for the company and the consumer, satisfying customer expectations efficiently; (3) allow decoupling the economic value of resource consumption and reduce environmental impact during the life cycle of products; and (4) imply an innovation in the corporate business model [8,18,19,20]. 

The implementation of the PSS supposes an increase of the efficiency in the operation and a reduction of the environmental impact, due to the reduction of resources consumed in the production phase eliminating traditional practices. At the end of the product life cycle, the company has the economic potential of reusing, in addition to the benefit of waste. The PSS increases the strategic positioning of the company, due to the added value perceived by customers in reducing costs, and the tax advantages and new international standards [21,22]. In short, the PSS allows the company to develop new markets; respond to the needs of consumers; build longer relationships with customers; and it can be a way to consolidate the corporate identity; although this is not always the case since, for example, companies that when transitioning to PSS offerings completely change their portfolio and therefore their offer should be considered, or the case of being a company focused on the product to be, in some cases, a company focused on services does not imply changing the corporate identity.

Although the PSS originates from a sustainability perspective, there has been a shift in focus from environmental benefits to economic benefits. PSS are increasingly seen as business strategies created by companies that seek to strengthen their market position and create a competitive advantage through transactional sales of non-traditional products.

PSS is often referred to as a model for improving the sustainability performance of traditional product systems, due to its potential to improve resource efficiency by extending product life and decoupling value from physical product delivery. However, PSS do not guarantee resource reduction or decoupling of resources [23,24,25,26]. From a corporate perspective, this is positive as it avoids cannibalization of the current market for new products but does not mean an overall reduction in resources.

In another order, the implementation of the PSS allows to reduce the gap between developed and undeveloped countries, since, in developed countries, more consumers and polluters, it implies the transformation to a service economy, while, in developing countries, the efficiency of the PSS improves the standard of living [27,28,29]. The PSS concept is directly associated with servitisation and functionality economy, and focuses on sustainability and CE, as well as enabling the development of the agriculture sector, among others.

### 2.1. Servitization

In previous decades, some companies focused solely on creating the product and outsourced the end customer service. However, this model opened a market for other companies to cover the entire cycle. Marketing researchers have called this process servitisation (SV) [30,31]. In industry, the concept of SV refers to the process of increasing the capabilities of a company to offer a greater experience for the end consumer.

Currently, the SV refers to claiming supplementary services for a product and offering them through the manufacturer, a key aspect for them to fulfil their growth plans. Although the services may be complementary to the main products, the implementation of the VS process has a direct impact on the company, since it involves reviewing the organizational structure, in addition to reflecting on the business processes or methods [30,32].

Proper application of the VS increases both earned income and budget stability by reducing dependence on sales. On the other hand, by increasing the loyalty rate, it confers a higher quality in the customer relationship model. The most relevant dimensions of SV are both the importance of tangible characteristics for the value of the offer, and the interaction between the manufacturer and the customer for the value of the offer [33,34].

The industry is in full transformation of SV, due to the irruption of manufacturers in the service sector. Therefore, in the industry there are levels of SV: provision of products; after sales services; and advanced services. The associated risk increases as these levels advance, since it increases when a customer pays for a service that occupies a certain time horizon, instead of if she/he pays for a product at a certain moment [35,36]. 

The services demanded by the SV process depend on the successful adoption of technology by the manufacturer, in direct relation to Industry 4.0 and the Internet of things (IoT). Hence, based on a product’s performance, companies can analyse effective behaviour and gain a better understanding of usage trends and future difficulties that may arise [37]. 

Among the benefits of SV is the development of long-term relationships between the manufacturer and the customer. Previously, manufacturers were simply the supplier of the product, whereas today they play an intrinsic role throughout the life of the product and beyond. Thus, the longer the duration of a customer relationship, the more profitable it will be for the manufacturer. Moreover, she/he considers that companies, in general, want to optimize the number of service providers they have to minimize the interruption of the processes, and the SV is a good option for the manufacturers [33,38,39].

### 2.2. Functionality Economics

Functionality economics (FE) seeks to add services to a product and consider changes in consumption by attending to the end user and economic models in more saving resources, producing benefits. The economic paradigm that underlies the FE refers to the fact that the value is in the benefits obtained from the use, in addition to the good or service itself and in the perception by the consumer, that is, in the value of work or of exchange. Thus, this model proposes to develop the use of the products more than their ownership, since, currently, companies do not sell only a product, but a function invoiced in relation to the use [40,41]. 

In this context, the FE supports the dematerialization of the economy by including costs in the final price, in addition to collaborating in the decoupling between economic activity and environmental impact. In the traditional economic scheme, producers create value and consumers destroy it through consumption. Whereas, in FE, the interests of both agents must coincide with the purpose of preserving, or creating, value. On the other hand, the digital revolution allows both the production and exploitation of the data generated from the use and the experiences of producers and consumers to form new resources and created values [42,43]. 

### 2.3. Sustainable Product-Service System 

PSS can create sustainable results, so if the model provides the sustainability advantage, it is called the sustainable product-service system (SPSS). This concept has emerged recently and is different from the ideas of cleaner production, eco-design and for the environment [44].

The concept not only refers to the environmental optimization of products and processes, but it requires radical-creative thinking to reduce environmental impacts, while maintaining an acceptable quality of service. SPSS are sociotechnical systems that can provide the essential end-use function of existing product, such as performance services, shared use, product life extension, or demand management [45,46]. 

SPSS create sustainable products in terms of environmental load and resource use, while developing product concepts as parts of sustainable comprehensive systems, providing a service to meet essential needs. This model looks for opportunities to innovate towards sustainability in the current global social, environmental and economic crisis and generate an alternative that significantly reduces the environmental impact of traditional production and consumption systems [15,21,47,48].

### 2.4. Product-Service System for a Circular Economy

PSSs focus on selling services and utilities rather than products. It has been labelled as a means or business model to carry out a CE, where economic growth is decoupled from the consumption of resources. However, the PSS is not an implied warranty for a CE. Consequently, CE strategies do not necessarily lead to decoupling economic growth from resource consumption in absolute terms. Resource untying occurs when resource use declines, regardless of the growth rate of the economic engine [49,50,51]. 

PSS can increase end-of-life reuse and recycling of products, increasing resource productivity, while minimizing waste generation. Despite the virtues of the PSS for the EC, it should be implemented with caution, since there are no tangible guarantees that it will reduce environmental impacts [52,53].

In CE-focused PSS, companies will have an incentive to extend the life of products and manufacture them as efficiently as possible in terms of costs and materials. Of the PSS classification according to their orientation (product, use and results), only those focused-on results have real contributions to resource efficiency and circularity. The circularity of the products and the efficiency in the use of resources is achieved with the implementation of results-oriented PSS, since in this the client will pay only for the provision of the desired results, which is also considered a service, and not for the consumer or for product ownership [54,55,56]. On the other hand, use-focused PSS could lead to less thorough use, making circularity difficult to produce; while those focused on the product, do not change the incentive to maximize product sales [55,57].

## 3. Materials and Methods

This study has carried out two reviews, one qualitative and the other quantitative, to achieve the stated objectives. The first, qualitative in nature, refers to a systematic review. It is a very widespread technique that collects information from research carried out by primary studies, in order to obtain a synthesis on a field of study [58]. This type of review is very common, so you can find research with systematic reviews from various disciplines [59,60,61], and from PSS specifically [62,63,64]. The second review that has been carried out, of a quantitative nature, is called the bibliometric analysis [65]. This analysis, widely used in different fields [51,66,67,68,69], allows us to identify the relative importance of publications, as well as the evolution and trends of a specific research field. 

The database chosen to support this research has been Scopus [70,71,72]. Key reasons behind the choice include easy accessibility, extensive repository of publications, and the ability to download a full record in RIS or CSV format for analysis with software tools. Among the researchers who choose this database to carry out bibliometric studies, are the authors [73,74].

The period selected for this study covers all scientific production on PSS, beginning in 2000 and ending in 2020. The data, downloaded and analysed in January 2021, represent all published research on PSS during the 21 years considered. The applied search, for which a total of 2560 investigations are obtained in the Scopus database, is defined by the parameters: (TITLE-ABS-KEY (“product/service system *”) AND PUBYEAR < 2021). However, in order to delimit the sample to be analysed, it was decided to include only the original articles, books, and book chapters. Therefore, the final search that defines the search is: (TITLE-ABS-KEY (“product/service system *”) AND DOCTYPE (ar OR bk or ch) AND PUBYEAR < 2021). This decision prevents double counting and duplication of results [75,76], since recent research supports the idea that conference papers, reviews and conference reviews only consider results published in other formats that are considered in this analysis. Thus, when applying this filter, the sample ends up obtaining a total of 1088 contributions to analyse.

The sample was downloaded in RIS format to filter duplicate records in references, author names or keywords, which could make the analysis difficult. Once this purification process was carried out, the variables analysed were as follows: annual publications, most prolific authors, institutions of affiliation of the most active authors, journals and countries, main thematic areas and keywords that define the trend in the research. This information is provided in the results section from tables and figures that indicate the most representative positions of each variable analysed.

**Figure 1:**
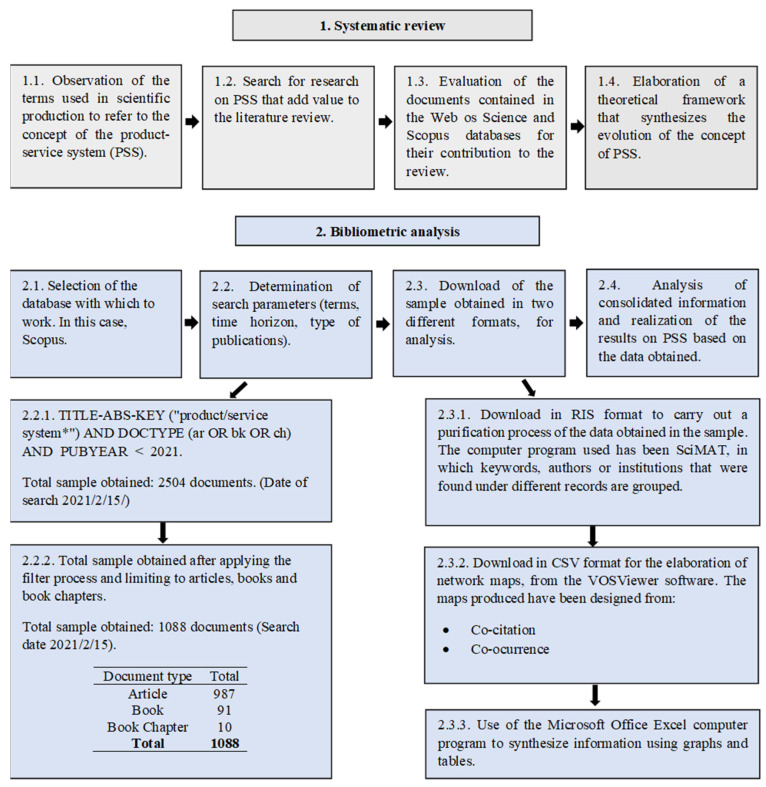
Explanation of the methodology used.

On the other hand, to graphically show international collaboration between the variables studied, network maps have been carried out. For its correct execution, the sample of 1088 documents was downloaded in CSV format and used in the VOSviewer tool [77], whose robustness for mapping scientific results has been proven [78]. Finally, to evaluate the relative importance of research in this area, several quality indicators were analysed, such as the number of citations received [79], the h-index [80,81,82] and the impact factor of the Scimago journal rank (SJR) [83]. In Figure 1 the stages of the process carried out are synthesized.

## 4. Results and Discussion 

### 4.1. Evolution of the Articles Ppublished

In this section, a general analysis of the scientific production oriented to PSS is carried out. The established time horizon begins in 2000 and ends in 2020, which makes it possible to cover all the articles, books, and book chapters made to date.

Table 1, which differentiates the analysis into three 7-year periods, shows the general growth trend experienced by this line of research. In the first period (2000–2006), a total of 31 contributions were published, compared to the 777 made during 2014–2020.

If reference is made to the authors who have published in the PSS line of research, from 2000 to 2006, a total of 49 authors are counted. In the second period, 2007–2013, this figure increases to 590 authors. Finally, in the last period, 2014–2020, the number rises to a total number of 1642 authors, a value that represents 78.90% of the total sample. The variable that relates the published research and the participating authors is the average number of authors, which goes from 1.6, in the first period, to 2.1, in the last two periods.

The countries also experience a general growth trend throughout the time horizon considered. From 2000 to 2006, 14 countries participated in this line of research. While, between 2014 and 2020, out of the 64 countries that make up the total sample of countries, 61 participated in this period. This means that in the last period analysed, only three countries (Liechtenstein, Venezuela and Israel) stopped publishing in this line of research.

Likewise, the growing interest in PSS is reflected in the number of citations. The first period (2000–2006) records 98 total citations, while the last period (2014–2020) reaches 26,143 citations. Regarding the number of journals, the first period (2000–2006) registers 10, while the last period (2014–2020) registers 259. Values that represent 3.13% and 80.98%, respectively, of the total number of journals (320) that publish the analysed scientific production. 

**Figure 2:**
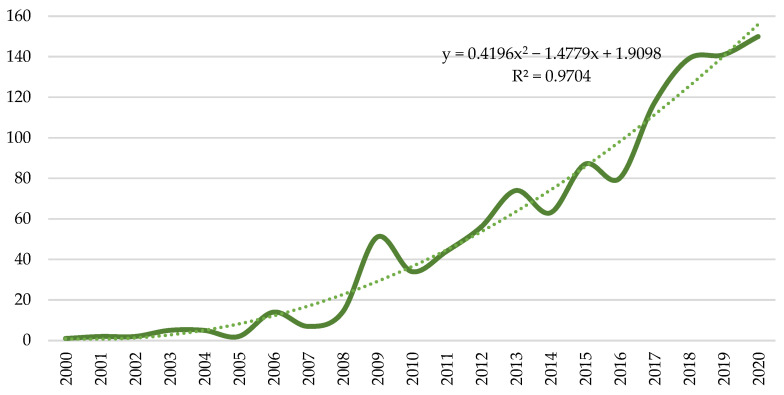
Number of documents published by year.

Figure 2 shows the documents carried out annually on PSS. The sample obtained throughout the period considered is a total of 1088 documents, of which 2.85% belong to the first period analysed, 25.74% to the second period and 71.42% to the last period. As of the year 2017, more than 100 contributions are registered annually, and the year 2020 stands out, for having the largest number of publications (150). If the research published annually is compared, the lowest percentage of variation (−60%) occurs in 2005, as the number of documents is reduced from five to two. On the contrary, the percentage of greatest variation is recorded in 2006, with a value of 600%, going from two publications to fourteen.

The first research, as the graph indicates, is from the year 2000. This publication, by Robin Roy (2000), makes a brief review of the sustainable measures that have been developed throughout history. However, as this author assures in his publication, all of them have limitations, such as the absence to be able to modify the production and consumption model, or the impossibility of achieving an environmentally sustainable situation, exclusively with these actions. In this context, the author mentions for the first time the concept of PSS, as an opportunity to modify consumption and production models, and have a direct impact on sustainability.

However, the research carried out by Tukker and Tischner (2006) clarifies that the benefits of PSS, such as the improvement of the value chain and the reduction of costs, occur because of good acceptance by the consumer and a correct decision-making on the service to be offered. In this way, these authors ensure that benefits can be affected in each case, and that the introduction of PSS does not always represent a competitive advantage. Hence, they suggest expanding research to ensure the sustainability and full utility of PSS.

In the second period analysed (2007–2013), the highest percentage of variation (803.2%) is registered in the number of articles, authors, countries, citations, and journals. It is interesting to highlight the term “servitization”, which refers to the change from the exclusive sale of products to the product/services system, and which was used for the first time in 2007.

In the considered time horizon, investigations are carried out that satisfactorily and sustainably integrate the PSS. For example, research by Geum and Park (2011), which provides theoretical and practical information on design and integration. In numerous investigations of this period, the difficulties that can arise when introducing PSS in a traditional product are considered. An example of this type of publication is that made by Cavalieri and Pezzotta (2012).

In the last period analysed (2014–2020), PSS are considered a combination that can help the transition towards a CE, and therefore, improve the life cycle of products [84]. However, it is not the only interest in this period analysed. Contributions such as those of Ingemarsdotter, Jamsin and Balkenende [85] present the IoT as a useful tool to be able to offer a complete PSS. Specifically, they propose the possibility of connecting the services offered to customers to the internet, in such a way that the information can be constantly updated by the providers.

In this context, numerous actions are published by the United Nations (UN) and the European Parliament with the aim of joining forces and contributing to a favourable change [86,87,88], although it is true that in the last two years analysed, the number of published investigations has stagnated. The trend as seen in the figure is positive, but the rate at which it is doing decreases significantly compared to previous years. In a way, this may be the precedent to a paradigm shift that includes new terms or interests in this field of study.

Currently there are various laws and regulations that try to respond to the changing needs of society and business models [89,90]. However, the low maturity of these concepts may be holding back the publication at the expense of the first results. However, the rise of this line of research is far from fading, as the challenges and research that are currently being posed remarkably fuel interest in finding effective solutions [91,92]. This evolution of the regulations and of the new terms identified in the scientific production will be expanded in the keywords section, since the main reasons that generate interest will be explained by periods

### 4.2. Analysis of Scientific Production by Subject Areas

**Figure 3:**
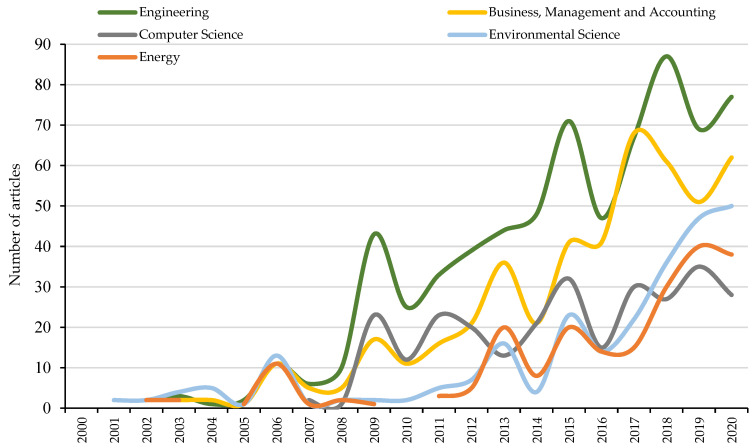
Comparisons of growth trends in subject areas in research by periods.

The Scopus database allows the analysed sample to be classified into up to a total of 27 categories [70]. In this case, the 1088 documents fall into 23 subject areas. Figure 3 graphically represents the evolution of the five categories with the highest representation. The main discipline is engineering with a total of 685 contributions, a value that represents 27.48% of the total sample. This category leads the rest of the disciplines due to the high number of contributions, since it has been considered annually throughout the time horizon analysed, except for 2000 and 2001.

In second place is business, management and accounting, with 475 publications (19.05%). This thematic area has been framed in research throughout the analysed period, except for 2001, which did not record any publication. The next discipline is computer science, with 11.35% and a total of 283 publications. In 2003, it registered an article, but it was not until 2007, when it began to be considered annually in publications. Environmental sciences, which is in fourth position, has 258 publications. This discipline represents 10.35% and is the only category that records publications (2) in 2001. Finally, energy is positioned as the fifth thematic area with 213 contributions, which represents 8.54% of scientific production on PSS. In total, these 5 thematic areas group 1914 publications, a value higher than that of the analysed sample (1088). This is because each research can be classified into one or more disciplines based on the interests of both the publisher and the authors themselves.

In Figure 3 the main categories have been represented, that is, those that gather more than 200 articles, and that, therefore, have a percentage of representation of the sample greater than 8%. Despite this, the categories that occupy the sixth position (decision sciences) and seventh position (social sciences), also group a high number of contributions with a total of 171 and 142 articles, respectively. Finally, the rest of the disciplines (16) do not reach 100 contributions or 4% representation.

Figure 3 allows to represent two changes experienced in the main subject areas (engineering and business, management and accounting). On the one hand, the beginning of the second period analysed (2007–2013), is defined by the exponential growth experienced in the publications framed in these two categories. The reason behind this event is the incorporation of new keywords, which had not been used before and which are directly related to these two themes. In fact, in the keywords section you will be able to know some of the terms incorporated from this second period analysed. 

On the other hand, between the years 2018–2019, there is a drop in the number of publications associated with these two main disciplines. There are two reasons for this situation. The first one is the reduction of publications made, together with an increase in the number of categories considered. This is because 139 contributions were published in 2018 and 141 documents in 2019. Moreover, to this variation of 1%, the consideration of new categories for the investigations carried out is added, since it goes from considering 14 disciplines in 2018, to referencing a total of 17 categories in 2019. Finally, the second reason that explains this reduction in the number of investigations for the two main categories, is the publication of regulations that prioritize other issues such as the CE or sustainability. That is why in the keywords section an analysis of the published European regulations will be made, as well as the main terms incorporated in each period analysed.

### 4.3. Distribution of Articles Published by Journal

The investigations developed can be published in a wide variety of journals, so they will be chosen according to the object of study and the perspectives of analysis that each one covers. In this way, Table 2 shows the 20 main journals that publish on PSS, which are focused on various topics: sustainability, production, manufacturing technology, industry marketing, etc. This table provides information on the main characteristics of the journals, such as the articles published, the country to which they belong, the h-index of the journal [93] or the quartile to which they belong in the SJR indicator [83]. Moreover, in relation to the published documents, the total citations received, the average number of citations per document or the publication period are detailed.

These 20 international journals represent 6.25% of the total of the journals that publish on PSS (320). However, they group together a total of 495 contributions, which represents 45.50% of the total sample analysed. This table is led by European journals, specifically those of British origin, since almost 50% belong to the United Kingdom (UK). Regarding the impact factor, all the journals, except for the *International Journal of Product Development*, belong to the second or first quartile, the latter standing out for representing 70% of the journals considered. 

All the journals have increased their publications throughout the time horizon, except for five of them that have a negative percentage of variation. In this context, the journal that occupies the second position, Sustainability, registers the greatest variation (5.10%) when going from 1 document between 2007–2013, to a total of 52 publications between 2014 and 2020. On the contrary, among the journals that experience a reduction in the number of published documents, the *Journal of Manufacturing Technology Management* stands out, going from 17 documents, in 2007–2013, to 6 publications, in 2014–2020, which translates into a variation of −65%.

The *Journal of Cleaner Production* tops the table. This journal belongs to the Netherlands and has the highest value in published documents (123), total citations received (8906), average citations per article (72.41) and h-index in research (48). It is positioned in the first quartile, registers an impact factor of 1866, and it is the only journal in the table that published a document in the first period analysed (2000–2006), which makes it a pioneer journal in this field of study. Moreover, this journal has published the most cited research of the entire analysed sample, with a total of 1214 citations, under the title “clarifying the concept of product—service system” [4].

The journal that follows is called *Sustainability*. This journal is of Swiss origin, has 53 publications, 404 total citations and an h-index of the journal of 68. It belongs to the second quartile, with an impact factor of 0.581, and its first research was published in 2013. The *International Journal of Production Research* has 42 publications and belongs to UK. This journal occupies the third position, however, it surpasses *Sustainability* in all the variables analysed, except in the number of publications made, since it has 1093 total citations, an average of 26.02 citations, an h-index of 20, and 125 in the journal. Furthermore, this journal has an impact factor very similar to the one that heads the table, and both are in the first quartile, unlike the second most prolific journal.

The *CIRP Annals Manufacturing Technology* journal occupies position 12. This journal, of American origin, despite having 13 documents, stands out for having the highest impact factor, with a value of 2544. In the opposite position, is the *Wt Werkstattstechnik* journal since it has the lowest value in all the variables in the table. This journal of German origin has 22 articles, but it has only 12 total citations, an average of 0.55 citations and an h-index in the publications of 2. Finally, the *Journal of Business Research*, in position 19, stands out for having the highest h-index in the journal. It has a value of 179 and even surpasses the journal that heads the table (*Journal of Cleaner Production*), which has an h-index in the journal of 173.

The diversity of categories studied by the most prolific journals stands out. The journal that heads the table, *Journal of Cleaner Production*, registers up to four different subject areas for the publications made on PSS. Specifically, there are publications on the disciplines of business, management and accounting, energy, engineering and environmental science. The same occurs for the rest of the sample, since if the study is extended to the three most prolific journals, a total of 6 disciplines are covered, of the 23 registered. These data reflect the multidisciplinary nature of PSS, as there is not only room for studies focused on a specific area. An example of this are the publications that consider PSS as an improvement for the life cycle of products and their performance. In this case, not only the economic results that could be generated are investigated (category of business, management and accounting and economics, econometrics and finance), but also, the environmental benefits that could cause (environmental science and earth and planetary sciences) and the processes that could ensure proper integration (decision sciences and computer sciences).

The following section mentions the scientific production carried out by the most prolific countries, institutions, and authors, who serve varied interests and disciplines.

### 4.4. Distribution of Articles Published by Author, Institution and Country

#### 4.4.1. Characteristics of the Main Authors

The total sample analysed has been carried out by a total of 2081 authors. Table 3 shows the 10 most prolific authors, representing 0.48% of the total sample analysed, but grouping a total of 180 documents. The table provides information on the number of documents produced by each author, the total number of citations received, the average number of citations per publication, the institution to which they belong or the h-index they have [82]. Finally, the date on which each author has published the first and last document is identified, which makes it possible to observe that all the authors are currently investigating in this line, except for two of them (Carlo Vezzoli y Rajkumar Roy).

European authors predominate, since they represent 80% of the table, and specifically the British, as this is the most frequently repeated nationality. Tomohiko Sakao, of Swedish origin, heads the table with a total of 33 publications and an h-index of 18, values higher than the rest of the authors in the table. This author, he published the first research of him in 2009 and belongs to Linköpings universitet. The British author, Fabrizio Ceschin, belongs to Brunel University London and is positioned in second place with 22 articles, 675 total citations and a h-index of 9. Rajkumar Roy, is the seventh author in the table, but stands out for its high value in citations received and an average of citations per document, with a value of 2182 and 145.47, respectively. This author from UK, belongs to City, University of London, and collaborated in the publication of “*State-of-the-Art in Product Service-Systems*” [94], which is positioned as the second most cited research, with a total of 1158 total citations. Finally, the Italian author Carlo Vezzoli stands out, for being the first author of the table to publish an investigation on PSS, specifically in 2006.

**Figure 4:**
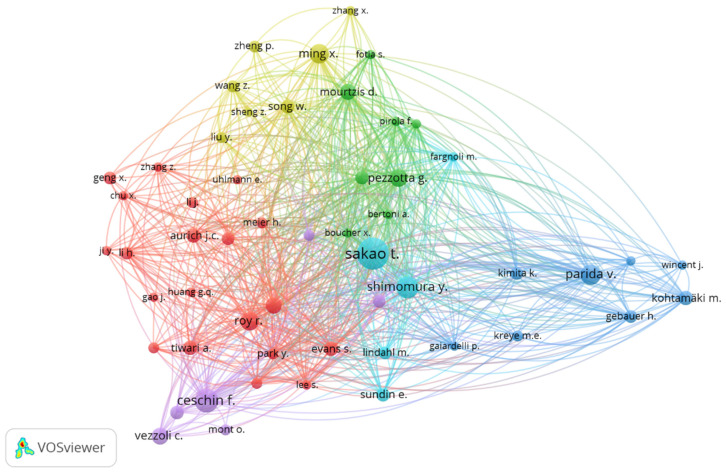
Network of cooperation based on co-authorship of the main authors.

After knowing the 10 most active authors in the PSS line of research, it is interesting to show a cooperation network based on co-authorship. Figure 4 graphically represents the relationship between authors, through the VOSViewer tool [77]. This analysis has been carried out for the 63 main authors, however, only 54 of them establish a relationship with others. The size of the circles indicates the number of documents published by each author, and the number of lines defines the collaborations made with other authors.

International cooperation is classified into six different clusters. The first one, red in colour, is made up of 20 authors and is led by the prolific author Rajkumar Roy. In this collaborative group, the authors Aurich, J.C. and Tiwari, A., both with 12 publications, and Evans, S. with 7 documents.

The second cluster (green) is led by the prolific authors Giuditta Pezzotta and Dimitris Mourtzis. The collaborative relationship in this group is established mainly with Italian authors such as Pirola, F., Terzi, S. and Fotia, S., all of them with seven published documents. An example of the research carried out by the authors of this second cluster is the publication “The Product Service System Lean Design Methodology (PSSLDM): Integrating product and service components along the whole PSS lifecycle” [95]. 

The third cluster, dark blue in colour, stands out for the close cooperation established between authors of different nationalities. In this case, the prolific author Vinit Parida, who leads the cluster, has the participation of authors such as Kimita, K., from Japan, Gaiardelli, P., from Italy, or Kreye, M. E., from Germany.

The fourth cluster, blue in colour, is led by the most prolific author of the analysed sample, Tomohiko Sakao. This Swedish author collaborates with 5 authors, including two highly recognized in this line of research, Shimomura, Y. and Sundin, E., with 20 and 11 publications, respectively. As a result of the collaboration between these authors, there is the article “Modelling Design Objects in CAD system for Service/Product Engineering” [96]. 

The fifth cluster is made up of seven authors and is represented in yellow. It is led by the prolific author Xingguo Ming, and has the collaboration of Song, W., Zheng, P. and Sheng, Z. 

Finally, the purple cluster is represented by the prolific authors Fabrizio Ceschin, and Carlo Vezzoli. The rest of the authors that make up this cluster are Sousa-Zomer, T.T., with 8 publications, and Diehl, J.C, with 11 documents and Kohtala, C. with 3 documents. As a result of their collaboration, numerous investigations are recorded [97,98] that are framed in this line of research.

#### 4.4.2. Characteristics of the Main Institutions

A total of 1896 institutions have contributed to the scientific production on PSS. Table 4 shows the 10 most prolific institutions based on the number of publications made. This table provides information on the country of origin, the total citations received, and the average of citations or the h-index of the institutions. On the other hand, to measure the international collaboration of these institutions, the collaboration index (in percentage), the total number of citations received for articles prepared internationally, and the total of citations received for articles that have not been published. 

All the institutions considered in the table are European, except for the Asian Shanghai Jiao Tong University. Once again, UK stands out for its high percentage of representation. The institution that heads the table is Linköpings Universitet, with a total of 57 publications, 1829 total citations, an average of citations of 32.09 and an h-index of 23. The second institution Shanghai Jiao Tong University occupies the second position with 51 publications, 873 total citations, and an average of 17.12 citations per article and an h-index of 17. However, the third institution in the table, Cranfield University, outperforms the previous institutions in total number of citations (3857), average of citations (77.14) and h-index (24). In fact, this institution is the one that published the research mentioned above as the second most cited [94]. The rest of the institutions in the table have values that range between 50 and 20 documents, between 500 and 1500 total citations, and an h-index of between 10 and 19.

In relation to the international collaboration carried out by these institutions, Vaasan Yliopisto stands out, due to its high rate (92.59%). This Finnish institution has produced 25 documents internationally and 2 nationally. Regarding the average of citations received, it has a value of 42.64 in international articles, and 2.50, in national articles. In the opposite position is the only Asian institution in the table, Shanghai Jiao Tong University. This Chinese institution registers a collaboration rate of 17.65%, since it has carried out 42 national and 9 international documents, with an average of 16.67 and 19.22 citations, respectively. Finally, among the 10 most prolific institutions, five of them have received more citations in articles with international collaboration, and another five have received more citations in articles without international collaboration.

#### 4.4.3. Identification of the Most Relevant Countries in Scientific Production on PSS

The production of 1088 documents has been carried out by a total of 64 countries. In Table 5, the 10 most prolific countries in the publication on PSS are considered, as well as their main characteristics; total citations, average of citations per document, h-index, or period in which the publications have been made. This table includes Asian, American, and European countries, although the latter predominate due to their high percentage of representation (60%).

The country that heads the table is UK with 200 publications. This country has 10,360 total citations, an average of 51.80 citations and an h-index of 49. UK was the first country in the table to publish research, making it the pioneer country. If the presence of this country is analysed in the variables analysed above, it is the country with the greatest presence both in journals, as well as in authors and institutions. The second place in the table is for China, with 194 publications, 2394 total citations and an h-index of 27. The first investigation of this Asian country was carried out in 2008, and currently, in the last period analysed (2014–2020) it has managed to position itself in the first position of the ranking with 154 documents with 34 more publications than UK. The last position in the table is occupied by Brazil with 44 publications, 1028 total citations, and an average of 23.36 citations and an h-index of 14. This country, despite starting its research career in the first period analysed (2000–2006), stands out for its absence in the tables of the most prolific institutions, journals, or authors.

Table 6 shows in detail the international collaboration carried out by the 10 most prolific countries. UK stands out for being the country with the highest number of collaborators, with a value of 37. Furthermore, it is among the main collaborators of all the countries in the table, except for Brazil. Among the main countries with which it cooperates, the presence of European countries stands out since of the 5 main ones, four are. The following countries with the highest number of collaborators are the Netherlands, with 29, Germany, with 26, and Italy, with 25. 

In relation to the collaboration index, Finland is positioned as the country with the highest value (77.42%) since it publishes 48 documents with the collaboration of other countries and 14 without international collaboration. With very similar values, USA follows, which has a collaboration rate of 70.91%, publishing 39 international and 16 national documents. On the contrary, Germany stands out, registering the lowest collaboration rate (25.83%). Finally, of the 10 countries considered in the table, 60% have the highest average of citations in publications made internationally.

**Figure 5:**
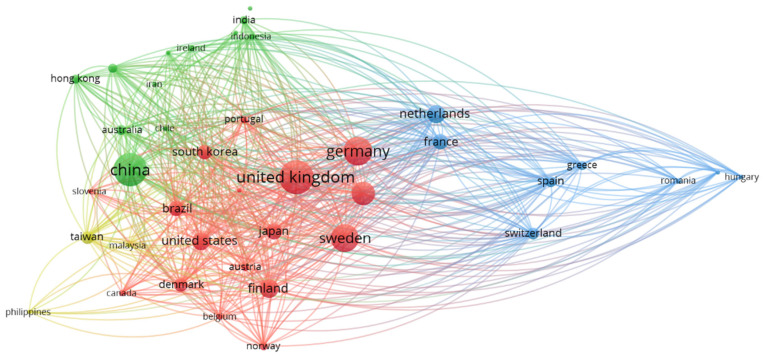
International cooperation based on curatorship between countries from 2000 to 2020.

Figure 5, through different clusters, graphically represents international collaboration between countries. The circles of each country vary depending on the number of publications made, and the lines established between countries symbolize cooperation. The first cluster, in red, is considered the most extensive, since it has the participation of 17 countries. It is led by UK, Germany, and Sweden, with the collaboration of countries such as USA, Denmark, Finland, and Brazil. This first cluster groups 904 documents and represents 85.44% of the total sample analysed. The second cluster, blue, is led by the Netherlands, and has the participation of France, Spain, Greece, and Switzerland, among others. In total it is made up of 8 countries, which manage to group 121 documents, that is, an 11.44% representation. The third cluster, green in colour, is led by China, and has the collaboration of other Asian countries such as India, Indonesia, and Hong Kong. Among all the countries that make up this cluster (12), they group a total of 279 documents, which represents 26.37% of the total sample obtained. The fifth cluster, in yellow, is made up of Taiwan, Malaysia, and the Philippines. This international collaboration group is the smallest, since it gathers 34 documents in total, and represents 3.21% of the analysed sample.

**Figure 6:**
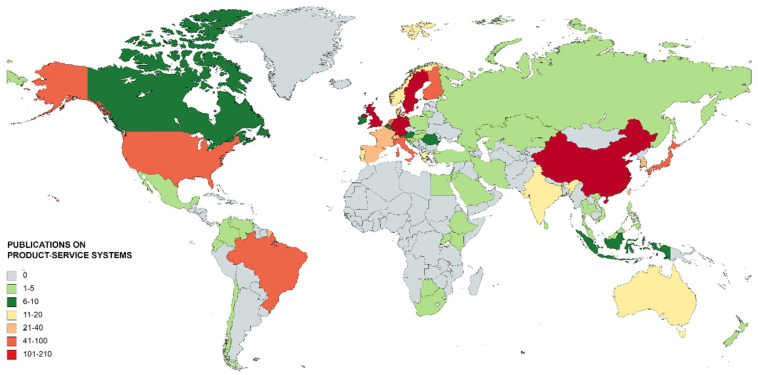
Worldwide publications on PSS.

Figure 6 represents the world map with the countries that have investigated PSS. This map provides information about the countries that occupy an advantageous position in the scientific production on PSS and those that still have research to do. The countries that have published between 1 and 5 documents stand out, as they are the largest bulk of the sample, since out of 64 countries, 33 belong to this group.

### 4.5. Keyword Analysis and Research Trends

Before analysing the keyword sample, a consolidation process was carried out to process minor variations in the terms obtained. Specifically, concepts were unified that, having the same meaning, were differentiated by their writing (accents, capital letters, hyphens, plurals, abbreviations, acronyms, etc.). After this process, 5475 keywords were registered with a total of 11,483 occurrences. The number of existing keywords is higher than the total number of documents analysed, since each investigation may use more than one term to correctly define the scope of study. 

In this section, information is first provided for each of the three periods analysed individually, and subsequently, the main keywords for the entire time horizon considered are presented. The first period analysed (2000–2006), has 31 documents and a total of 231 keywords. The terms “product development”, “sustainability”, “environmental impact”, “sustainable development” and “life cycle” stand out for registering the highest number of occurrences. Research from this period focuses on finding alternatives that can improve future sustainability, as well as reduce the excessive use of natural resources or edit consumption and production patterns. In this context towards an environmental improvement, numerous measures and action plans are published by the European Union that help to achieve these objectives. In Table 7, some of these actions approved during the analysed period are mentioned.

A methodology based on the lexicon used has been carried out through the search for keywords that contain the concepts of sustainability, environment, natural resources, etc., in the regulations. The measures presented by the European Union, to deal with sustainability problems, are completed by the research carried out in this first period analysed, thus demonstrating the direct relationship established. In this way, while the keywords are used in the investigations, the regulations set objectives that seek to change unsustainable consumption habits, improve sustainable production, avoid the production of large amounts of waste, and pursue a responsible use of renewable natural resources, improve the performance of production processes, consider the complete life cycle of products, or improve waste management.

The second period analysed (2007–2013) registers new regulations and agreements that promote sustainable actions by companies and consumers, at the European, national, regional, and local level (see Table 8). After carrying out a lexical analysis of the regulations of this new paradigm, new challenges are presented to face, such as the need to be more energy efficient in production processes, promote the design of products with fewer resources, improve the potential environmental performance of the industry itself or seek competitive advantage in the efficiency of the PSS itself.

The keywords with the highest number of occurrences in this second period analysed are “product design”, “life cycle”, “innovation”, “industry” and “customer satisfaction”. The number of investigations in this period (2007–2013) amounts to 280 and the number of terms used in these publications reaches 1961 terms. It is interesting to mention the existence of research that has been financed by the European organizations themselves, with the aim of finding solutions to the challenges of the moment. Within the Community Support Framework 2007–2013, programs such as “The Framework Program for Research, Technological Development and Demonstration (2007–2013)” [105], these have been in charge of financing research projects related to numerous topics.

On the other hand, it is pointed out that, in this second period, the investigations carried out by non-European countries (China, USA, Japan, etc.) begin to be registered among the main positions, so there may be a slight variation between some European regulations and what is established globally and, therefore, the interests developed in scientific publications. Therefore, Table 9 provides information on the main events worldwide, which affect all countries that are part of the scientific production on PSS.

In the third period analysed (2014–2020), new terms were used, reaching a total of 3966 keywords. In this case, the terms with the highest number of occurrences in the investigations are “servitisation”, “decision making”, “circular economy”, “sales” and “competition” (see Table 10). From the perspective of legislation, the objectives focus, among others, on strengthening the decisions taken, taking advantage of the new opportunities of the circular and collaborative economy to complete the PSS, expanding the policy frameworks with binding objectives, achieving an integrated approach to the production-consumption systems, or investing in environmental technology and ecological innovation [51,110]. The importance of the measures carried out at the international level is highlighted, since as can be seen, the regulations promote research and the development of new possibilities.

To continue with the analysis of the complete time horizon, in Table 11, the 20 main keywords of all the research carried out are represented. These refer to 0.37% of the total keywords but represent 13.8% of the occurrences made. It is clarified that the terms that have been applied in the search for this study itself, such as “product-service system” or “PSS”, have not been considered in the table.

Between 2000 and 2006, 40% of the words that are currently considered keywords in research had not been used in any study. Some of these terms were: design, supply chain, business models, or industrial research. In the second period, these keywords began to be considered relevant for this field of research, going from 0 occurrences to a minimum of 10, except for the term CE, which was only mentioned in one document.

Finally, in the last period analysed (2014–2020), all keywords began to be used frequently in publications, until they were considered as the terms with the most occurrences in the line of research on PSS.

Table 11 is headed by the term product design. In the 21 years analysed, it has appeared in a total of 218 documents, of which 162 occurrences belong to the last period analysed (2014–2020). The second term, with a total of 156 documents, is manufacture. This keyword stands out for being the term in the table that has experienced the highest percentage of variation between the first period (2000–2006) and the second period (2007–2013), since it went from 1 occurrence to 37. The term that occupies the tenth position, CE, stands out as the keyword that has experienced the greatest variation between the second and third periods. This is because it went from being mentioned in 1 research, between 2007–2013, to having 60 occurrences, between 2014–2020, which reflects the recent relationship that is being established between the term of PSS and the CE.

**Figure 7:**
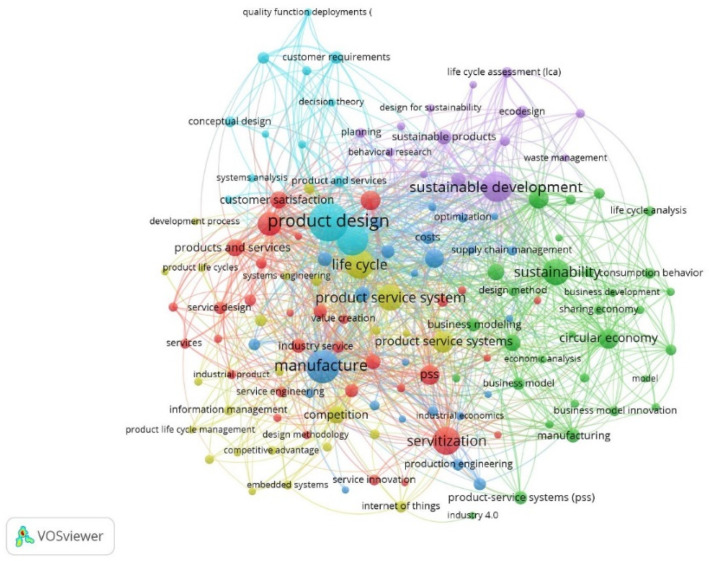
Main keywords network based on co-occurrence from 2000 to 2020.

Figure 7 represents the keyword network based on co-occurrence. Each cluster groups together a set of terms that generally refer to the same object of study, therefore, in this case, 6 different areas of interest can be distinguished.

The first cluster, in red, is represented by the terms products and services, customer satisfaction, servitisation, and value creation. The keywords used are related to the new business concept that the industry is focusing on, which is intended to offer customers a complete service that goes beyond the mere sale of products. This is reflected in the creation of value for the business model itself and an increase in customer satisfaction.

The second cluster, in light blue, is led by terms such as product design, conceptual design, costumer requirements and quality function deployment. This set of terms refers to the technical part of PSS, which must meet a series of quality standards and respond to the functions that the client himself expects from the system that is offered.

Third, the dark blue cluster is made up of the concepts of manufacture, optimization, costs, supply chain management and production engineering. This cluster represents the investigations that contemplate the improvement in its production chain or in the optimization of costs, which are benefits of implementing the PSS correctly.

The fourth cluster, yellow, is represented by the terms life cycle, system engineering, competitive advantage and development process. The terms included in this cluster are related to the very line of research that is being analysed, that is, the possibility of positively influencing the competitiveness of the industry through the offer of a product/service system that allows expanding the cycle of lifetime.

The green cluster is led by the concepts of CE, economic analysis, business development, business modelling and business model innovation. This set of terms establishes a relationship between the PSS and the economic part. It could be said that in this cluster the costs of its introduction into the industry are considered, as well as the sales or benefits that would be obtained.

Finally, the purple cluster refers to the sixth group of terms. In this case, scientific production focuses on the possibility of positively influencing the environmental situation through the PSS themselves. An example of the issues that are considered in the research is that of enjoying a service without having to physically own the product that offers said desired service, so this new paradigm could generate new benefits both for the life cycle of the products as well as for the environment. The terms used in this last cluster are sustainable development, waste management, eco-design, or life cycle assessment, among others.

**Figure 8:**
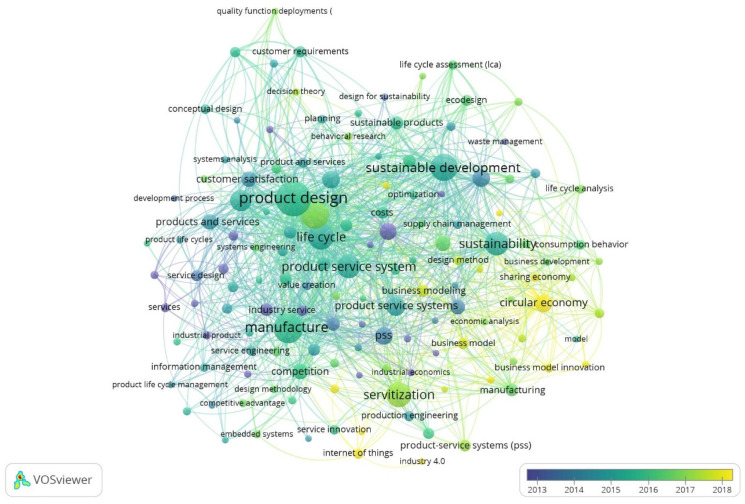
Product-services systems keywords over time.

To complete the analysis of keywords, Figure 8 shows the period in which each term has been incorporated into scientific production. The concepts are arranged in the temporal space from 2013 to 2018, and vary in colour, from dark blue to yellow, depending on the maturity of the term itself.

The first concepts that appeared in this line of research are development process, service design, business model and optimization, as has been seen in the objectives of European regulations. This group of words refers to the beginning of a new concept that needs to be investigated to answer the doubts that arise about whether it is sustainable, improves competitiveness, or optimizes the production process.

The concepts that emerge in the second period, around 2015, are eco-design, competitive advantage and supply chain management. These terms refer to the PSS as a reality in which the consequences of introducing it in the industry are beginning to be valued, as well as the need to invest in environmental technology, or technological innovation. Finally, the concepts of sharing economy, Industry 4.0, IoT and CE have been the last to be introduced in this field of research. In this last period, scientific production focuses on assessing the benefits from its offer in the market, as well as the possibility of combining different existing tools, such as the collaborative economy, IoT or the advancement of technology, to improve benefits.

**Figure 9:**
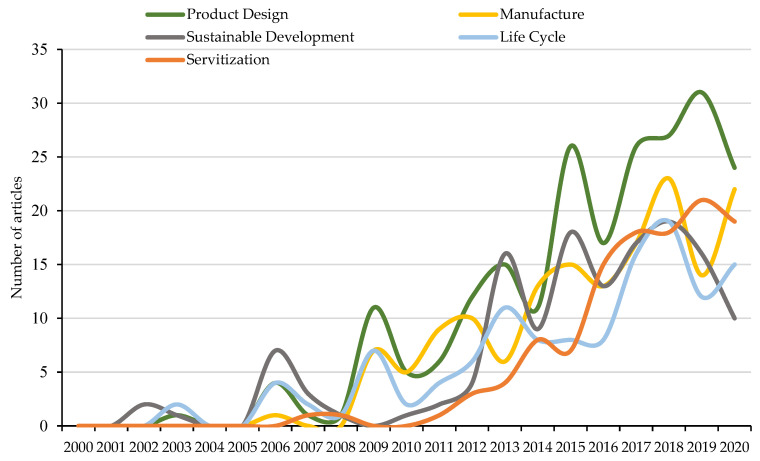
Evolution of the five main keywords.

Chronological analysis provides information about the approximate date of incorporation of keywords into publications. However, the high volume of concepts used throughout scientific production makes it difficult to visualize the evolution of the main terms. Therefore, Figure 9 shows the five keywords with the most occurrences, as well as the number of publications in which they have been considered throughout the period studied.

The five keywords with the highest number of occurrences are those at the top of Table 11, specifically product design, manufacture, sustainable development, life cycle and servitisation. This analysis allows knowing the terminological preferences in the scientific production of PSS and the presence of these terms for each year analysed.

The first term, product design, has a total of 218 occurrences throughout the 21 years analysed and is represented in green. The first occurrence dates back to 2003 under the investigation “Product-Service Systems, A Perspective Shift for Designers: A Case Study—The Design of a Telecentre” [113], although it is from 2006 when it begins to register occurrences annually. The highest percentage variation is registered in 2009, since it has 11 occurrences compared to 1 in 2008. 

The second keyword that heads this classification is manufacture, with 156 occurrences and represented in yellow. The first time this term was used was in a publication made by the *Journal of Cleaner Production*, specifically in 2006 [114]. As of the year 2009, this concept begins to be considered annually in scientific publications, and the greatest percentage variation, with a value of 117%, occurs in 2014, from 6 occurrences to 13. 

The third key phrase considered in the Figure 9 is sustainable development. This term is represented in grey and has a total of 138 occurrences. The first year in which this term was mentioned was in 2002 with a total of two occurrences, both investigations being published in the *Journal of Cleaner Production* [4,115]. The highest percentage variation (300%) in the number of occurrences was recorded in 2013, from 4 occurrences to 16. 

The fourth term considered is life cycle. This keyword, which is represented in blue, has a total of 125 occurrences. The first two documents that referenced this term were published in 2003 [116,117], and it was as of 2006 when it was considered annually in the PSS investigations. This term registers its highest percentage variation with a value of 600%, in 2009, since it goes from one occurrence to seven. Servitization is the keyword that occupies the fifth position by the number of occurrences (117 records). In the Figure 9 is represented in orange, and its first occurrence can be seen in a 2007 publication [94]. Since 2011, it is considered annually in the investigations, and the highest percentage of variation (200%) occurs in 2012, going from one occurrence to three. Finally, 2012 stands out as the year in which the five keywords analysed began to gain special relevance in the scientific production of PSS.

Table 12 presents the top five terms used by the most prolific institutions. To obtain these keywords, a debugging process has previously been carried out to eliminate those concepts that, although having a high value in the number of occurrences, were terms that did not provide information (priority journal, article, research, investigation, etc.) or that referred to the analysed search itself (product-service systems, PSS). 

The main keywords for each institution make it possible to differentiate the lines of research carried out by each of them. Institutions such as Delft University of Technology focus their research on the design of a product that addresses an environmental dimension, using concepts that refer to sustainable development or the introduction of environmental costs (eco-costs). Other institutions, such as Luleå tekniska Universitet, focus more on the technical side of design, having terms such as manufacturing, product development, or product design. Although many interests can be differentiated, since other institutions are registered that are inclined towards business models with terms such as decision making, sales or customer requirements. It stands out that all prolific institutions, except for Vaasan Yliopisto, register among the five keywords, the term product design. Finally, none of the institutions has geographical concepts among the most used terms for PSS publications.

**Figure 10:**
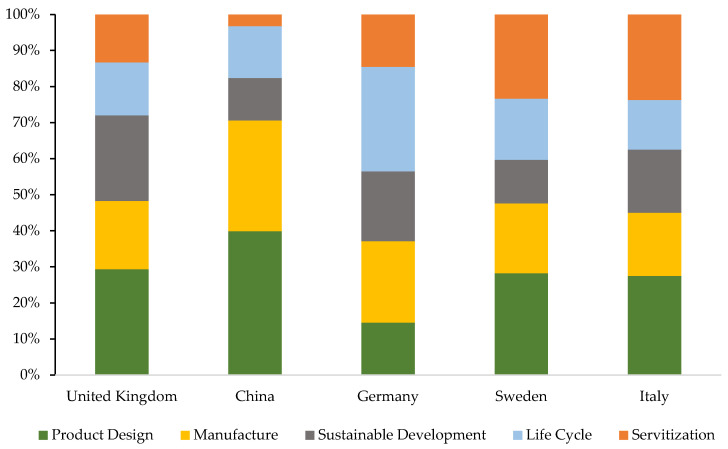
Main PSS keywords for the most prolific countries.

To conclude with this section, Figure 10 represents the percentage of repetition represented by the keywords in the investigations of the five most active countries. The product design term is the one that receives the highest percentage of repetition for all countries, except for Germany, which registers the highest percentage in the life cycle term. The lowest percentage for the three most prolific countries is received by the fifth term (servitisation), while for the fourth and fifth countries, the lowest percentages are registered by sustainable development and life cycle, respectively. China, with 39.87%, is positioned as the country that registers the highest percentage of repetition, specifically for the term product design (39.87%). Furthermore, this same country registers the lowest percentage of repetition, specifically in servitisation with 3.27%. In this way, the figure graphically represents the presence of each of the main terms, with the aim of knowing to what degree they are considered in the investigations of each country.

### 4.6. Relationship between PSS and the Terms of Sustainability and CE

At the beginning of the study, the objective of knowing the link between PSS and the concepts of sustainability and CE was raised. That is why the results must conclude with a study of the evolution of both terms in the analysed sample. To carry out this section, the investigations that refer to these study areas have been selected in the 1088 documents analysed. The interrelation between both terms is total since the objective of the CE is to extend the useful life of the products and therefore contribute favourably to sustainability. However, this last term has a more general nature in PSS, since investigations can be directed towards respectful materials, processes or even regulations that facilitate their progress.

**Figure 11:**
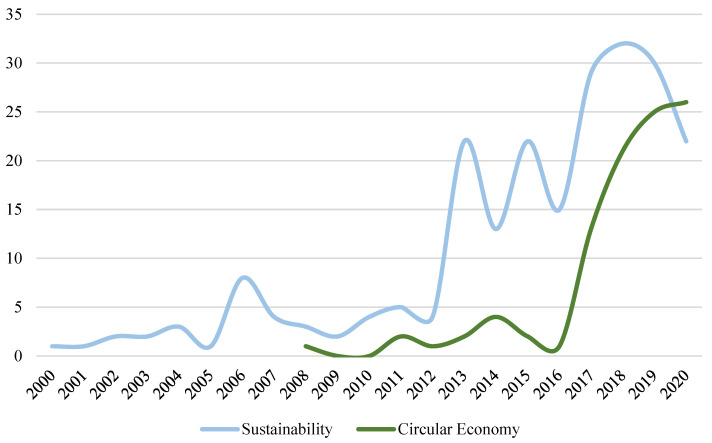
Relationship between PSS and sustainability and CE terms.

A total of 225 documents on sustainability and 98 publications on CE have been obtained, after the purification process. Figure 11 shows the publications for each field of study throughout the time horizon considered. It can be seen how the concept of PSS originated from a sustainable perspective, since they were used together from the beginning of the research (year 2000), making clear the direct relationship between both terms. On the other hand, after the filtering process that only obtains research on sustainability, there is a decrease in the number of researchers associated with PSS in the years 2019 and 2020. 

In this case, the values reflect a percentage variation of −6% and −27%, respectively. These last two years analysed are directly related to the other subject studied (CE). This term begins to gain special relevance from 2017, in which a variation of 1200% is registered when going from one contribution to thirteen. Only publications on this subject are seen from 2008, so it hints at the recent interest. In fact, if this figure is compared with the network map of keywords (Figure 8), it will be possible to contrast the information and affirm the low maturity of the term.

Table 13 indicates the main characteristics for CE and sustainability research, in the field of PSS. The comparison between both lines of research allows us to identify common variables such as the prolific author, Tomohiko Sakao, the Linköpings universitet and Delft University of Technology, or the *Journal of Cleaner Production* and *Sustainability* journals. However, the path of each line of research conditions the main characteristics. For this reason, the table synthesizes the main features of each one of them, to be able to establish comparisons.

**Figure 12:**
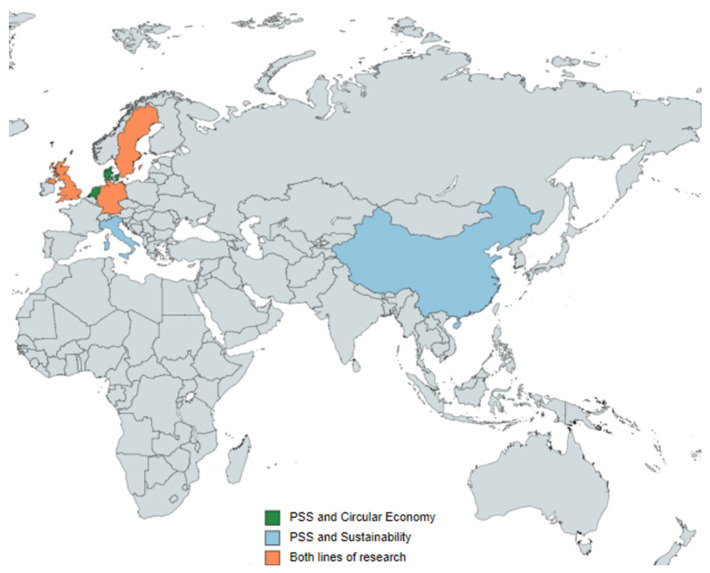
Most prolific countries for each line of research.

In the geographical area, scientific production is produced mainly by European countries. However, one Asian country is identified among the most active in research on PSS and sustainability. For this reason, Figure 12 is constructed to illustrate the five most prolific countries for each line.

After knowing the evolution of both terms in the PSS research, the analysis is completed with the keywords that both contributions represent. Table 14 summarizes some of the terms most used in scientific production.

**Figure 13:**
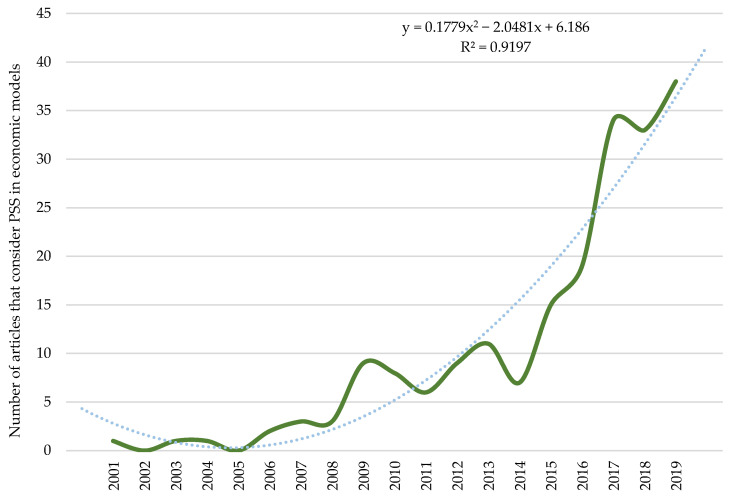
Research on the economic dimension of PSS.

As mentioned at the beginning of this study, the multi-disciplinarity of this term favours the introduction of numerous thematic areas that contribute knowledge to research on PSS. Therefore, to conclude the results section, special mention is made of the new approach that has been given to economic benefits within the PSS line of research. Figure 13 represents the increasing trend in the quantity of research being published on economic benefits, business opportunities and increased competitiveness when considering PSS in economic models.

After analysing the path of this line of research, it is still pending to know the future results associated with the new terms incorporated into PSS. New business models that incorporate the concepts of CE or ecological innovation, among many others, may favour the introduction of PSS and with it, the improvement of the results obtained so far. 

### 4.7. Discussion

The results of the bibliometric analysis illustrate the exponential growth that has occurred in publications, since in the last five years 547 contributions have been reached, which represents 50.28% of the total sample. This is supported by the growing interest in presenting alternatives to traditional consumption models that are environmentally responsible as well as competitive [1,2,3]. In fact, there are currently different areas of study associated with PSS, such as sustainability, SPSS [44]. Regarding the beginning of this line of research, the first publication that mentioned the term PSS dates from 2000, written by Roy. At present, with a high percentage of variation in the number of publications, the sample reaches 1088 contributions. This expansion in the scientific production of PSS may be due, in addition to the importance that is given at present, to the multidisciplinary nature of the subject, since the studies require not only technical knowledge, but also environmental or economic knowledge, among others [21,40].

The two main thematic areas, which stand out for their percentage of representation compared to the analysed sample, are engineering and business, management and accounting with 685 and 475 publications, respectively. In fact, this is confirmed by the European regulations analysed, which register periods with a high commitment by the industry, innovation and product design [108] and other periods with a high interest in CE, competition and sales [112]. On the other hand, in the ranking of the twenty most prolific journals, their relative importance stands out, since they all belong to the first and second quartiles, except for *International Journal of Product Development*, which is in the third quartile. Among the sample of journals analysed, *Journal of Cleaner Production* holds an advantageous position compared to the rest of journals, since it leads the ranking in the three periods analysed. Its success is due, among other reasons, to the publication of the most cited article in the analysed sample, which clarifies and defines the concept of PSS [4]. Even so, the *Journal of Business Research* and *CIRP Annals Manufacturing Technology* are also given prominence, for surpassing the h-index and impact factor, respectively, of the journal that heads the ranking.

The number of authors participating in this line of research amounts to 2081, being Tomohiko Sakao, with 33 contributions, and Fabrizio Ceschin, with 22, the most prolific. In this classification, the pioneer of this term, Roy R., has the highest value of citations (2182) and average of citations (145.47), compared to the rest of the authors. In order of importance, the institutions Linköpings Universitet and Shanghai Jiao Tong University are classified, having 57 and 51 publications, respectively. However, the institution that occupies the third position, Cranfield University, surpasses the previous two in citations received, average of citations and h-index.

In geographical terms, a greater involvement is detected at the global level, since it goes from 14 countries active in the research, to a total of 61 countries for the last period analysed. Specifically, UK and China stand out for having a positive impact on PSS research and the highest values in publications. Finally, the countries that register the highest collaboration, with values of 77.42% and 70.91%, are Finland and USA.

The study reveals that the main keywords used throughout the scientific production on PSS are: product design, manufacture, sustainable development, life cycle and servitisation [114,115,117]. Said concepts have been addressed at the beginning of the research and are considered representative in the field of study. Finally, the realization of network maps establishes the main links between these terms, and mentions the areas of interest investigated, highlighting the two most representative concepts currently, CE and sustainability. These data reveal the growing expansion of the field of study to other disciplines, allowing for the expansion of concepts and the correct delimitation of the direct relationship established with each of the terms studied.

## 5. Conclusions

The aim of this article is to examine the dynamics of global research on the PSS business model and related community legislation so far. The study is carried out from a systematic review and bibliometric analysis, in which the period 2000–2020 is considered as the time horizon. In the general field, variables such as the main countries, authors or keywords that make up the field of study are identified. In the field, there are two lines of research (sustainability and CE) that are included in the analysed sample, and with which a direct relationship is established. In this case, the research trajectory of both topics is represented, and the most representative characteristics of each line are specified.

The results indicate that the scientific production on PSS has evolved throughout the analysed period, according to the interests of the moment, which is the reason why this line of research can be defined by a multidisciplinary nature. Likewise, special mention is made to the link it has with the concepts of sustainability and CE, since the analysis carried out represents the exponential growth that has been taking place in the last nine years. Finally, in order to clarify future research trends, the recent interest that is being given to the economic variable is mentioned. In this sense, it is essential to mention the importance of future political actions, nationally and internationally, since they will be in charge of directing the proper introduction of PSS from an economic and sustainable perspective. Therefore, future research could focus on aspects such as (1) analysing how approved policies affect the introduction of PSS, (2) delineating the environmental and economic interests that are related to PSS to be used by policy makers or (3) analysing qualitatively and quantitatively if the introduction of PSS as an alternative to traditional consumption models, notably benefits the closest environment.

On the other hand, this study has some limitations. First, the results addressed in this research have been obtained using quantitative techniques, thus it could be completed with additional information obtained from other qualitative or quantitative tools. Second, the analysis has been carried out from the investigations registered in the Scopus database. In this sense, the study could be completed with research from other databases, because although Scopus is one of the most extensive repositories, it does not cover all scientific production on PSS. In this way, the introduction of new research could provide a more comprehensive analysis of current trends in PSS research. Third, this paper has incorporated the exact concept of “product-service system*” into the search parameters. However, there are numerous issues related to this term, such as innovation, industrialization, sustainability and digitization in product-service systems. Therefore, an analysis that covers all areas of interest could yield revealing results on the main interests in this line of research. Finally, to obtain the sample, the scientific production focused on PSS was decided to limit to publications that were original articles, books or book chapters. However, it would be convenient to introduce conference paper in the sample, since it is true that some recent results may be considered in this type of document, and not yet in articles. Therefore, such research could be included, being previously compared with the other three types of documents (original articles, books and book chapters) to ensure that they do not constitute research already covered in the sample.

To conclude this study, it is worth clarifying that the main contribution of this study has been to show the latent interest in the scientific community in presenting alternatives to traditional consumption models that degrade the environment and the immediate surroundings. Hence, this analysis has been related in parallel to the regulations published at European and global level to establish a clear and direct relationship. 

## Figures and Tables

**Table 1:** Major characteristics of PSS from 2000 to 2020.

Period	A	AU	C	TC	TC/A	J
2000–2006	31	49	14	98	3	10
2007–2013	280	590	36	3886	14	97
2014–2020	777	1642	61	26,143	34	259

A = articles per period; AU = number of authors; C = number of countries, TC = total citations in articles; TC/A = total citations per article; J = number of journals per period.

**Table 2:** The most active journals in PSS from 2000 to 2020.

Journal	A	TC	TC/A	Hi-a	Hi-j	SJR (Q)	C	R (A)		
								2000–2006	2007–2013	2014–2020
*Journal of Cleaner Production*	123	8906	72.41	48	173	1.886 (Q1)	Netherlands	1 (16)	1 (17)	1 (90)
*Sustainability*	53	404	7.62	12	68	0.581 (Q2)	Switzerland	0	93 (1)	2 (52)
*International Journal of Production Research*	42	1093	26.02	20	125	1.776 (Q1)	UK	0	4 (14)	3 (28)
*CIRP Journal of Manufacturing Science and Technology*	32	834	26.06	16	44	1.193 (Q1)	Netherlands	0	3 (14)	5 (18)
*Jisuanji Jicheng Zhizao Xitong Computer Integrated Manufacturing Systems CIMS*	27	177	6.56	8	31	0.288 (Q2)	China	0	6 (11)	6 (16)
*Journal of Manufacturing Technology Management*	23	1235	53.70	16	65	1.173 (Q1)	UK	0	1 (17)	22 (6)
*Wt Werkstattstechnik*	22	12	0.55	2	9	0.270 (Q2)	Germany	0	8 (9)	8 (13)
*IFAC Papersonline*	20	225	11.25	7	63	0.332 (Q2)	Austria	0	0	4 (20)
*International Journal of Advanced Manufacturing Technology*	20	631	31.55	13	112	0.999 (Q1)	UK	0	5 (11)	14 (9)
*Advanced Engineering Informatics*	14	146	10.43	8	75	0.946 (Q1)	UK	0	0	7 (14)
*Computers in Industry*	14	750	53.57	13	93	1.007 (Q1)	Netherlands	0	7 (9)	25 (5)
*CIRP Annals Manufacturing Technology*	13	935	71.92	11	143	2.544 (Q1)	USA	0	9 (8)	24 (5)
*Computers and Industrial Engineering*	13	321	24.69	7	121	1.469 (Q1)	UK	0	23 (2)	10 (11)
*International Journal of Operations and Production Management*	12	820	68.33	10	129	2.187 (Q1)	UK	0	16 (3)	15 (9)
*International Journal of Production Economics*	12	462	38.50	9	172	2.379 (Q1)	Netherlands	0	0	9 (12)
*Industrial Marketing Management*	11	492	44.73	7	125	2.084 (Q1)	Netherlands	0	49 (1)	13 (10)
*International Journal of Product Development*	11	148	13.45	7	25	0.238 (Q3)	UK	0	11 (7)	31 (4)
*International Journal of Product Lifecycle Management*	11	78	7.09	6	20	0.374 (Q2)	UK	0	26 (2)	16 (9)
*Journal of Business Research*	11	180	16.36	8	179	1.871 (Q1)	Netherlands	0	0	11 (11)
*Production Planning and Control*	11	258	23.45	9	70	1.394 (Q1)	UK	0	0	12 (11)

A = number of articles; TC = total citations for all articles; TC/A = number of citations by article; Hi-a = h-index articles; Hi-j = h-index journal; SJR = Scimago journal rank (Quartile); Q = quartile; C = country; R(A) = rank position by the number of articles published; UK = United Kingdom; USA = United States of America.

**Table 3:** The most prolific authors in PSS research during 2000-2020.

Authors	A	TC	TC/A	Institution	C	1st A	Last A	h-Index
Sakao, T.	33	989	29.97	Linköpings universitet	Sweden	2009	2020	18
Ceschin, F.	22	675	30.68	Brunel University London	UK	2009	2020	9
Shimomura, Y.	20	466	23.30	Tokyo Metropolitan University	Japan	2009	2020	10
Parida, V.	18	839	46.61	Vaasan Yliopisto	Finland	2014	2020	14
Ming, X.	17	214	12.59	Shanghai Jiao Tong University	China	2013	2020	8
Pezzotta, G.	15	504	33.60	Università degli Studi di Bergamo	Italy	2009	2020	9
Roy, R.	15	2,182	145.47	City, University of London	UK	2007	2019	12
Vezzoli, C.	14	361	25.79	Politecnico di Milano	Italy	2006	2018	7
Durugbo, C.	13	282	21.69	University of Liverpool Management School	UK	2010	2020	9
Mourtzis, D.	13	193	14.85	Panepistimion Patron	Greece	2016	2020	8

A = number of articles; TC = total citations for all articles; TC/A = number of citations by article; C = Country.

**Table 4:** Characteristics of the most outstanding institutions.

Institution	C	A	TC	TC/A	h-Index	IC (%)	TCIC	TCNIC
Linköpings Universitet	Sweden	57	1829	32.09	23	56.14%	35.38	27.88
Shanghai Jiao Tong University	China	51	873	17.12	17	17.65%	19.22	16.67
Cranfield University	UK	50	3857	77.14	24	36.00%	68.44	82.03
Luleå tekniska Universitet	Sweden	45	1407	31.27	20	53.33%	39.04	22.38
Delft University of Technology	Netherlands	39	1526	39.13	15	51.28%	38.90	39.37
Politecnico di Milano	Italy	33	684	20.73	13	51.52%	24.76	16.44
Vaasan Yliopisto	Finland	27	1071	39.67	16	92.59%	42.64	2.50
University of Cambridge	UK	24	1479	61.63	14	37.50%	18.22	87.67
Loughborough University	UK	23	559	24.30	13	34.78%	10.13	31.87
Università degli Studi di Bergamo	Italy	22	770	35.00	11	45.45%	32.10	37.42

C = country, A = number of articles; TC = total citations for all articles; TC/A = total citations per article; IC = percentage of articles made with international collaboration; TCIC = number of citations in articles with international collaboration; TCNIC = number of citations in articles without international collaboration.

**Table 5:** The most relevant countries in number of articles.

Country	A	TC	TC/A	h-Index	1st A	Last A	R (A)		
							2000–2006	2007–2013	2014–2020
UK	200	10,360	51.80	49	2000	2020	1(8)	1(72)	2(120)
China	194	2394	12.34	27	2008	2020	0	3(40)	1(154)
Germany	151	5117	33.89	32	2006	2020	6(2)	2(60)	4(89)
Sweden	141	5471	38.80	35	2002	2020	2(7)	4(37)	3(97)
Italy	99	2111	21.32	27	2006	2020	7(2)	6(16)	5(81)
Finland	62	2182	35.19	24	2004	2020	5(2)	16(3)	6(57)
Netherlands	56	3617	64.59	19	2001	2020	3(6)	8(13)	9(37)
USA	55	1583	28.78	19	2003	2020	9(2)	7(16)	7(37)
Japan	50	672	13.44	14	2006	2020	8(2)	5(20)	13(28)
Brazil	44	1028	23.36	14	2006	2020	11(1)	12(6)	8(37)

A = number of articles; TC = total citations for all articles; TC/A = number of citations by article; 1st A = First article; Last A = Last Article; R = rank position by the number of articles published.

**Table 6:** International collaboration of the most prolific countries.

Country	NC	Main Collaborators	IC (%)	TC/A	
				IC	NIC
UK	37	China. Italy. Finland. Sweden. Germany.	39.50%	43.94	56.93
China	23	UK. Hong Kong. France. Sweden. Singapore.	32.47%	18.52	9.37
Germany	26	UK. Italy. Sweden. Netherlands. USA.	25.83%	68.90	21.70
Sweden	21	Finland. UK. Germany. Japan. Switzerland.	43.97%	34.15	42.46
Italy	25	UK. Germany. Netherlands. Sweden. Finland.	39.39%	22.41	20.62
Finland	19	Sweden. UK. Switzerland. USA. China.	77.42%	41.25	14.43
Netherlands	29	UK. Italy. Germany. France. South Africa.	55.36%	55.65	75.68
USA	20	Sweden. China. Finland. Germany. UK.	70.91%	37.03	8.69
Japan	12	Sweden. USA. UK. Finland. Netherlands.	40.00%	18.45	10.10
Brazil	8	Italy. Netherlands. Portugal. Denmark. Germany.	34.09%	14.60	27.90

NC = number of collaborators; IC = percentage of articles made with international collaboration; TC/A = number of citations by article; IC = international collaboration; NIC = no international collaboration.

**Table 7:** Legislation of the European Union from 2000 to 2006.

2000–2006
European Climate Change Program [87]
Sustainable Development in Europe for a Better World: European Union Strategy for Sustainable Development [88]
Green Book [99]
Sixth European Community Action Program on the Environment [100]
EU Action Plan to Boost Environmental Technologies for Innovation, Growth and Sustainable Development [101]
Report of the European Environment Agency [102]
Thematic strategy on the sustainable use of natural resources [103]
Strategy for Sustainable Development [104]

**Table 8:** Legislation of the European Union from 2007 to 2013.

2007–2013
Seventh Framework Program from 2007 to 2013 [105]
Energy Policy for Europe [106]
The Lisbon Community Program: Proposal for 2008–2010 [107]
Action Plan for Sustainable Consumption, Production and Industry [89]
Report of the European Environment Agency [102]
Roadmap to a Competitive Low-Carbon Economy in 2050 [90]
Energy Efficiency Plan [108]

**Table 9:** Events carried out by the UN.

2000–2020
World Summit on Sustainable Development [109]
Paris Agreement—United Nations Convention on Climate Change [86]
2030 Agenda for Sustainable Development—Sustainable Development Goals [91]

**Table 10:** Legislation of the European Union from 2014 to 2020.

2014–2020
Report of the European Environment Agency [102]
Next Steps for a Sustainable European Future—European Action for Sustainability [111]
The European Green Deal [92]
New Circular Economy Action Plan [112]

**Table 11:** Main keywords from 2000 to 2020.

Keyword	2000–2020		2000–2006		2007–2013		2014–2020	
	A	%	R (A)	%	R (A)	%	R (A)	%
Product Design	218	20.0%	6 (5)	16.1%	1 (51)	18.2%	1 (162)	20.8%
Manufacture	156	14.3%	140 (1)	3.2%	2 (37)	13.2%	2 (118)	15.2%
Sustainable Development	138	12.7%	4 (10)	32.3%	5 (26)	9.3%	4 (102)	13.1%
Life Cycle	125	11.5%	5 (6)	19.4%	3 (33)	11.8%	5 (86)	11.1%
Servitisation	117	10.8%	0	0.0%	28 (10)	3.6%	3 (107)	13.8%
Sustainability	111	10.2%	2 (12)	38.7%	17 (15)	5.4%	6 (84)	10.8%
Decision Making	92	8.5%	0	0.0%	18 (13)	4.6%	7 (79)	10.2%
Sales	86	7.9%	195 (1)	3.2%	4 (27)	9.6%	9 (58)	7.5%
Environmental Impact	63	5.8%	3 (11)	35.5%	21 (11)	3.9%	11 (41)	5.3%
Circular Economy	61	5.6%	0	0.0%	526 (1)	0.4%	8 (60)	7.7%
Product Development	59	5.4%	1 (12)	38.7%	15 (17)	6.1%	14 (30)	3.9%
Design	58	5.3%	0	0.0%	14 (17)	6.1%	10 (41)	5.3%
Customer Satisfaction	49	4.5%	25 (2)	6.5%	9 (20)	7.1%	15 (27)	3.5%
Competition	48	4.4%	70 (1)	3.2%	25 (10)	3.6%	12 (37)	4.8%
Products and Services	46	4.2%	0	0.0%	7 (22)	7.9%	21 (24)	3.1%
Innovation	45	4.1%	117 (1)	3.2%	10 (19)	6.8%	18 (25)	3.2%
Supply Chains	36	3.3%	0	0.0%	29 (10)	3.6%	17 (26)	3.3%
Business Models	35	3.2%	0	0.0%	13 (17)	6.1%	32 (18)	2.3%
Sustainable Products	32	2.9%	214 (1)	3.2%	47 (7)	2.5%	22 (24)	3.1%
Industrial Research	32	2.9%	0	0.0%	19 (13)	4.6%	30 (19)	2.4%

**Table 12:** Top five keywords for the top ten institutions.

Institution	Keywords				
	1	2	3	4	5
Linköpings Universitet	Product Design	Manufacture	Life Cycle	Servitisation	Sales
Shanghai Jiao Tong University	Decision Making	Product Design	Manufacture	Sales	Customer Requirements
Cranfield University	Manufacture	Product Design	Industrial Research	Business Models	Integrated Products
Luleå tekniska Universitet	Servitisation	Manufacture	Product Design	Product Development	Digitalization
Delft University of Technology	Product Design	Sustainable Development	Sustainability	Circular Economy	Eco-costs
Politecnico di Milano	Product Design	Sustainable Development	Life Cycle	Design for Sustainability	Design Methodology
Vaasan Yliopisto	Servitisation	Manufacture	Business Modelling	Digitalization	Manufacturing
University of Cambridge	Product Design	Servitisation	Business Model Innovation	Life Cycle	Manufacture
Loughborough University	Product Design	Sustainable Development	Circular Economy	Economics	Availability
Università degli Studi di Bergamo	Product Design	Service Engineering	Life Cycle	Sales	Simulation

**Table 13:** Main characteristics for CE and Sustainability research.

PSS and Sustainability		
Author	Institutions	Journal
Ceschin, F.	Delft University of Technology	*Journal of Cleaner Production*
Sakao, T.	Linköpings universitet	*Sustainability*
Vezzoli, C.	Brunel University London	*International Journal of Production Research*
Diehl, J.C.	Universidade Federal de Santa Catarina	*Journal of Industrial Ecology*
Sousa-Zomer, T.T.	Politecnico di Milano	*Ecological Economics*
**PSS and CE**		
**Author**	**Institutions**	**Journal**
Sakao, T.	Linköpings universitet	*Journal of Cleaner Production*
McAloone, T.C.	Cranfield University	*Sustainability*
Pigosso, D.C.A.	Delft University of Technology	*Resources Conservation and Recycling*
Amasawa, E.	The University of Tokyo	*Journal of Industrial Ecology*
Boks, C.	Loughborough University	*Business Strategy and The Environment*

**Table 14:** Main keywords for sustainability and CE research.

PSS and Sustainability	PSS and CE
Sustainable Development	Sharing Economy
Design for Sustainability	Collaborative Economy
Sustainable Business Models	Collaborative Consumption
Eco-design Principles	Circular Business Model
Eco-efficient Services	Circular Design
Environmental Impact	Circular Economies
Ecology of Commerce	Circular Solution
Waste Management	Circular Strategies
Sustainable Products	Circular Supply Chains
Ecological Impact	Circularity Strategy
Eco-efficient Value Creation	Closed Loop Lifecycle Management
Environmental Sustainability	Closed-loop Design
Eco-innovation	Closed-loop Product Lifecycle Management
Sustainable Consumption	Closed-loop Supply Chain

## Data Availability

Data were obtained from Elsevier′s Scopus database (www.scopus.com (accessed on 17 September 2021)).
